# Proprioceptors of the human pericardium

**DOI:** 10.1007/s00395-024-01075-9

**Published:** 2024-08-09

**Authors:** Lea M. Piermaier, Svenja Caspers, Christina Herold, Michael Wolf-Vollenbröker, Patrick Brzoska, Eric Bechler, Timm J. Filler

**Affiliations:** 1 Institute for Anatomy I, Medical Faculty & Hospital Düsseldorf, Heinrich-Heine-University, Building 22.02, Floor U1, Room 15, Universitätsstraße 1, 40225 Düsseldorf, Germany; 2 Institute of Neuroscience and Medicine (INM-1), Research Center Jülich GmbH, Jülich, Germany; 3 Medical Faculty & Hospital Düsseldorf, Cécile and Oskar Vogt Institute of Brain Research, Heinrich-Heine-University, Düsseldorf, Germany; 4 Department of Diagnostic and Interventional Radiology, Medical Faculty, University Hospital Düsseldorf, Heinrich-Heine-University, Düsseldorf, Germany; 5 Core Facility for Magnetic Resonance Imaging, Medical Faculty, University Hospital Düsseldorf, Heinrich-Heine-University, Düsseldorf, Germany

**Keywords:** Pericardium, Proprioception, Ruffini-like corpuscles, Distribution pattern

## Abstract

In the human organism, all functions are regulated and, therefore, require a feedback mechanism. This control involves a perception of the spatial tensile state of cardiac tissues. The presence and distribution of respective proprioceptive corpuscles have not been considered so far. Therefore, a comprehensive study of the entire human fibrous pericardium was conducted to describe the presence of proprioceptors, their density, and distribution patterns. Eight human pericardial specimens gained from our body donation program were used to create a three-dimensional map of proprioceptors in the pericardium based on their histological and immunohistochemical identification. The 3D map was generated as a volume-rendered 3D model based on magnetic resonance imaging of the pericardium, to which all identified receptors were mapped. To discover a systematic pattern in receptor distribution, statistical cluster analysis was conducted using the Scikit-learn library in Python. Ruffini-like corpuscles (RLCs) were found in all pericardia and assigned to three histological receptor localizations depending on the fibrous pericardium’s layering, with no other corpuscular proprioceptors identified. Cluster analysis revealed that RLCs exhibit a specific topographical arrangement. The highest receptor concentrations occur at the ventricular bulges, where their size reaches its maximum in terms of diameter, and at the perivascular pericardial turn-up. The findings suggest that the pericardium is subject to proprioceptive control. RLCs record lateral shearing between the pericardial sublayers, and their distribution pattern enables the detection of distinct dilatation of the heart. Therefore, the pericardium might have an undiscovered function as a sensor with the RLCs as its anatomical correlate.

## Introduction

Proprioception serves the unconscious and conscious perception of connective tissues’ tensile state and motion. The term “proprioception” was introduced in 1906 by Charles Sherrington [60], who differentiated this sense emanating from the organism itself from “exteroception” and “interoception”. Even if interoception is not clearly defined and conceptualized, it is rooted in the vegetative system and is currently mainly discussed for its influence on the psyche [13], whereas the proprioceptive system is based on peripheral corpuscular receptors. These corpuscles are mechanoreceptors of the deep sensitivity that serve the body's self-awareness in space. Proprioceptors include various sensory structures, such as Ruffini-, Vater-Pacinian-, and Golgi-Mazzoni corpuscles, Krause end bulbs, Golgi tendon organs, and free nerve fibers. These register different stimuli, such as stretching, tension, vibration, or shearing. To date, the scientific literature on proprioceptors in both animals and humans has primarily focused on the musculoskeletal system [7, 12, 51]. Basically, mechanosensing is also present in the internal organs, and proprioceptors are already described for the pancreas [8, 22, 26, 63] and the thymus [71]. There is a lack of published research on corpuscular receptors for the pericardium. However, these sensors could be relevant in the sensory consideration of tamponade, pain, pericardial inflammation, or heart enlargement pathophysiology. It is challenging to obtain information for the assignments of the different sensors, e.g., physiological conduction of a single corpuscle, as neither their presence nor their exact location and distribution is known. Even the publications describing cortex-evoked responses only assume which mechanosensory receptors' specific impulse patterns are recorded [43, 53].

The fibrous pericardium consists of three sublayers of collagenous connective tissue interwoven with elastic fibers [17, 29, 30, 73]. Contrary to the common conception that the pericardium only operates under high diastolic volumes, it naturally couples diastolic ventricular pressure–volume relationships [24]. Movement and extension of the human pericardium depend on the amount of synovial filling of the pericardial gap, ranging between 15 and 60 ml [36, 72], and the dimension of the heart. The pericardial cavity not only enables gliding motion but also facilitates the transmission of mechanical stimuli to the parietal pericardium.

Whereas proprioceptive sensation is mediated by the somatic nervous system, the myocardium itself is supplied only by autonomic fibers [35, 38, 66, 70]. Kostreva and Pontus describe mechanosensory afferents via the phrenic nerve [37]. Still, the source of these sensorics in the pericardium remains unclear. Animal studies indicate that the phrenic nerve contains sensory components making up 30–45% of its composition [39, 40, 47]. This sensory portion of the phrenic nerve may transmit propriosensory afferents. Such afferents are discussed to play a role in pericardial pain perception [25, 76]. However, the specific receptors responsible for this nociception are unknown.

Normally, corpuscular proprioceptors are present in all connective tissue, and the human heart possesses an evident collagenous exoskeleton known as the pericardium. Furthermore, embryologic malformations with congenital absence of the pericardium can cause notable cardiac dysfunction, such as abnormalities in the conduction system, arrhythmias, and chest pain [23, 48, 49, 52, 55–57, 59, 67]. The severity of the pathology depends on the type and extent of the congenital absence. This implies that the pericardium maintains normal cardiac function beyond its mechanical functions, which have been considerably summarized by different authors [30, 33]. On the other hand, a missing pericardium does not necessarily lead to mechanical problems of the heart itself [15, 16]. Since the pericardium may not impact the heart's mechanical function, it could serve an additional function. Given the reported sensation of pericardial pain, it may be involved in perceiving sensitive impulses. We, therefore, aimed to identify the neural feedback components of the human pericardium.

To determine whether the pericardium functions as a receptor to control cardiac dynamics, we investigated the presence and types of proprioceptors occurring in the human pericardium. Receptors may be associated with the histological organization of the fibrous pericardium and could display a potential topographic system (i.e., density and distribution pattern) throughout the pericardium.

## Methods

### Body donation

Human pericardial tissue was obtained from eight body donors participating in the body donation program of the Heinrich-Heine-University Düsseldorf (HHU). The heart of a ninth female body donor was taken to create the volume-rendering and surface-rendering models of the “standard-pericardium”. The selected specimens had to cover the following conditions:

1. No surgical procedures near the heart or mediastinum/within the chest;
2. No known or visible heart disease;
3. No pacemaker or other interventions; and
4. No macroscopic primary tumor or metastasis in the mediastinal area

This study was approved by the Ethics Committee of the medical faculty of the University of Düsseldorf with the number 2021–1565, adhering to the following criteria: Declaration of Helsinki and written consent for the use of body materials for study and research purposes.

### Macroscopical sectioning

The entire thoraces of eight human cadaveric donors (four female and four male specimens) were fresh-frozen at – 79 degrees Celsius for at least 1 week. Horizontal sections of equal thickness (10 mm) were drawn along the entire heart length from cranial to caudal for each specimen. Since Ruffini-like corpuscles (RLCs) can grow up to several millimetres in length, we chose a circumferential evaluation of the pericardium from cranial to caudal at these intervals to avoid double-counting of the same RLC. The obtained sections underwent free-floating fixation in 4% formaldehyde (MORPHISTO^®^) for 2 weeks. Afterwards, the pericardium with adjacent structures was harvested from each section around the entire heart circumference for further histological processing.

### Tissue processing and staining

**Fig. 1:**
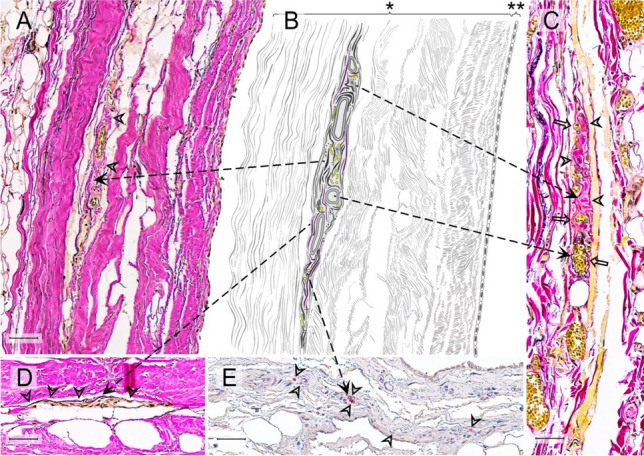
Histological criteria of Ruffini-like corpuscles (RLCs) in the human pericardium. Histological criteria of these receptors are the presence of meandered vessels and scattered nerve fibers, the occurrence of pale epitheloid cells, a minor density of collagenous fibers compared to the surrounding area, and demarcation from the environment by straightened elastic fibers. Since not all criteria of an RLC are always in one histological plane, we show a variety of receptors to demonstrate all previously mentioned criteria. All fusiform structures within the pericardium that fulfill at least three of the histological criteria were counted as RLC. **A** Histological image (section thickness: 7 μm; stain: Elastica van Gieson; scale bar: 50 μm) of an RLC in the pericardium of an 80-year-old male donor. The arrowheads indicate the less dense collagen fibers within the receptor than the surrounding area. **B** Schematic drawing of the RLC shown in A) with all histological criteria depicted. The arrows point to the corresponding criteria in the histological images. **C** Histological image (section thickness: 7 μm; stain: Elastica van Gieson; scale bar: 50 μm) of an RLC in the pericardium of an 86-year-old female donor. The arrowheads direct to the epitheloid cells, and the arrows to the meandering vessels. **D** Histological image (section thickness: 7 μm; stain: Elastica van Gieson; scale bar: 50 μm) of an RLCs in the pericardium of an 80-year-old deceased man. The arrowheads indicate elastin fibers, which demarcate the receptor from the surrounding tissue. **E** Histological image (section thickness: 7 μm; scale bar: 50 μm) with immunochemical staining (pan-Neurofilament) of a RLC of an 83-year-old female donor. The arrowheads point to the receptors, specifically stained scattered nerve filaments. * = pericardium fibrosum, ** = lamina parietalis pericardii serosi

In order to identify the number and location of the proprioceptors, the pericardial samples were processed using paraffin histology. The orientation of the slides was perpendicular to the surface to differentiate the pericardial sublayers. The sections were sliced at a thickness of 7 μm and stained with Elastica von Gieson staining to identify RLCs. Additional immunohistochemistry in subsequent sections exemplarily verified the plausibility that the corpuscles counted in the histochemical staining were Ruffini corpuscles. The distribution of nerve fibers and the occurrence of free Schwann cells were set as criteria. For this purpose, primary Antibodies against pan-Neurofilament (pNF; BioLegend [# 837904]) and Myelin Basic Protein (MBP; BioLegend [# 836504]) were used at concentrations of 2 μg/ml (pNF) and 1 μg/ml (MBP). To further confirm the diagnosis of Ruffini corpuscles, antibodies against S100 (Dianova [# DNA-AB-179523]; dilution: 1/200), a marker for sensory nerve fibers, Calcitonine Gene-Related Peptide (CGRP; abcam [# AB135271]; concentration: 15 mg/ml), a peptide released by certain sensory fibers in the peripheral nervous system, Transient Receptor Potential Channel 4 (TRPC4; Invitrogen [# PA5-18987]; concentration: 5 mg/ml), a non-selective calcium channel of sensory fibers, and tyrosine hydroxylase (Merck Millipore [# MAB318]; concentration: 12 μg/ml), to demonstrate the sympathetic-efferent innervation of RLCs, were applied. Secondary antibodies (anti-mouse IgG, VECTOR [# BA-2000] and anti-mouse IgM, BioLegend [# 401102] at a concentration of 1:250 for pNF and anti-mouse IgG, VECTOR [# BA-2000] at a concentration of 1:300 for MBP) and streptavidin HRP (BioLegend [# 405210]) at a concentration of 1:800 were used to label the NovaRED dye (VECTOR [# SK-4800]). The detected neuronal supply ensures that the corpuscle is equipped with the expected neuronal components, i.e., scattered nerve fibers within the RLCs along the longitudinal axis of their fusiform shape (Fig. 1). The positive finding of MBP was used to assign the pale epithelioid cells to Schwann cells.

### Data collection

The histological samples were scanned with a slice scanner (Motic EasyScan Infinity 100) and digitally analyzed using the QuPath software (Version 0.2.3[6]). It is generally accepted that RLCs cover the following histological criteria and are graphically summarized in Fig. 1:

- Spindle-shaped receptor arrangement
- Occurrence of pale epithelioid cells
- A lower density of the collagen fibers compared to the surrounding tissue,
- And demarcation from the surroundings by straightened elastic fibers
- Vessels meandering through the corpuscle

Histologically visible topographic proximity to nerves or nerve ingrowth into the corpuscles can be considered an additional criterion.

All structures within the pericardium that fulfilled three or more of the histologic criteria in addition to the shape were counted as RLCs.

### Statistics

To detect any systematics in the distribution of RLCs, we used cluster analyses that analyzed the density of corpuscles per area according to the relative coordinates of the three-dimensional pericardial surface. This analysis was conducted using the Scikit-learn library [19] in Python, while the Plotly library was used for 3D scatter plots [1].

With the help of hierarchical agglomerative clustering with WARD linkage, a dendrogram was created to partition the data logically [50, 74]. The resulting dendrogram displays the distance between merged clusters to facilitate detecting equitable partitions.

In addition to the hierarchical approach, we applied mean shift clustering to identify local maxima of neighborhood densities [10]. For each data point, the algorithm calculates the center of mass within a window surrounding that point. The window is then shifted towards this new center. When the need for shifting ceases, the iteration stops, and the data point is assigned to the identified center. Data points assigned to the same center form a cluster, and the center represents the point of the highest density.

When estimating the window size, which has a significant impact on the number of clusters found, the bandwidth estimation function available in the scikit-learn library was utilized. We then employed the density information to generate a heatmap, where each data point is colored in accordance with its relative distance from its corresponding center.

### Visualization

A surface rendering and a volume rendering model were created to visualize the distribution and arrangement of proprioceptors in the human pericardium. These three-dimensional models were imaged from a heart, including the pericardium. For this purpose, a female body donor was fixated with 4% formaldehyde (MORPHISTO®) in its entirety. All tissues surrounding the pericardium were precisely removed to preserve the heart, together with its contiguous suspension and diaphragm, in their natural in situ arrangement. An epoxy frame maintained the natural diaphragmatic protrusions caused by the liver and stomach into the thoracic cavity. This heart, used as the “standard-pericardium”, then underwent scanning using magnetic resonance imaging and photogrammetry.

#### MRI

A 3D whole heart (with pericardium) MRI dataset was used for the volume rendering model in order to visualize the heart's internal and to match the eight pericardia to the "standard pericardium". Volume rendering was performed by specifying the color and opacity for each voxel based on its image intensity using 3D Slicer (https://slicer.readthedocs.io/en/latest/user_guide/modules/volumerendering.hl). Data acquisition for the three-dimensional volume rendering model was performed on a 3 T scanner (Magnetom Trio, Siemens AG, Healthineers, Erlangen, Germany) using a 12-channel head coil. Data of the heart was acquired using a 3D Magnetization Prepared Rapid Gradient Echo (MP-RAGE) sequence with the following parameters: echo time (TE)/ inversion time (TI)/ repetition time (TR) = 3.35/900/1720 ms; flip angle = 9°; acquisition matrix = 384 × 384 × 256; voxel size = 0.5 × 0.5 × 0.5 mm^3^; number of averages = 5; acquisition time = 29:12 min.

#### Photogrammetry

The surface rendering model for visualizing the statistical outcomes was generated using photogrammetry. More than 1000 photos were taken from all angles of the “standard pericardium” using a reflex camera (model: Canon 750D with APS-C sensor, lens: 60 mm macro). We followed Spiriev et al.’s [62] digitization process. In brief: the images were transferred to photogrammetry software, which generated a dense point cloud that was used to calculate a coordinate system. Next, a three-dimensional mesh was created, and textures were overlaid. Finally, the finished 3D model was converted to Blender software.

Mapping and visualization of the proprioceptors were accomplished by digitally transferring all receptors found onto the volume rendering and surface rendering model of the "standard pericardium" using the 3D-slicer software (Version 5.1.0[21]). Each receptor position was determined as precisely as possible based on the histological findings, considering the adjacent environment and anatomical landmarks.

#### Image presentation

The images were digitized using a slice scanner (Motic EasyScan Infinity 100) and corresponding software. Image processing, including white balancing, contrast correction, noise reduction, and cropping, was performed using Affinity Photo (version 2.3). Image composition and labeling were created using Affinity Designer (version 2.3).

The utilization of large language models served to ascertain the grammatical and syntactical correctness of the text, as well as to identify alternative terms or phrases.

## Results

A total of eight fibrous pericardia were examined for the presence and distribution of proprioceptors. RLCs were found in large numbers at all pericardia. However, no Vater-Paccinian- or Golgi-Mazzoni-corpuscles, Krause end bulbs, or Golgi tendon organs were found in any specimens. In all eight pericardia, we found a sum of 1476 RLCs. The number of receptors is much higher when considering the total surface of the pericardium, as opposed to solely analyzing our selected 10 mm intervals.

**Fig. 2:**
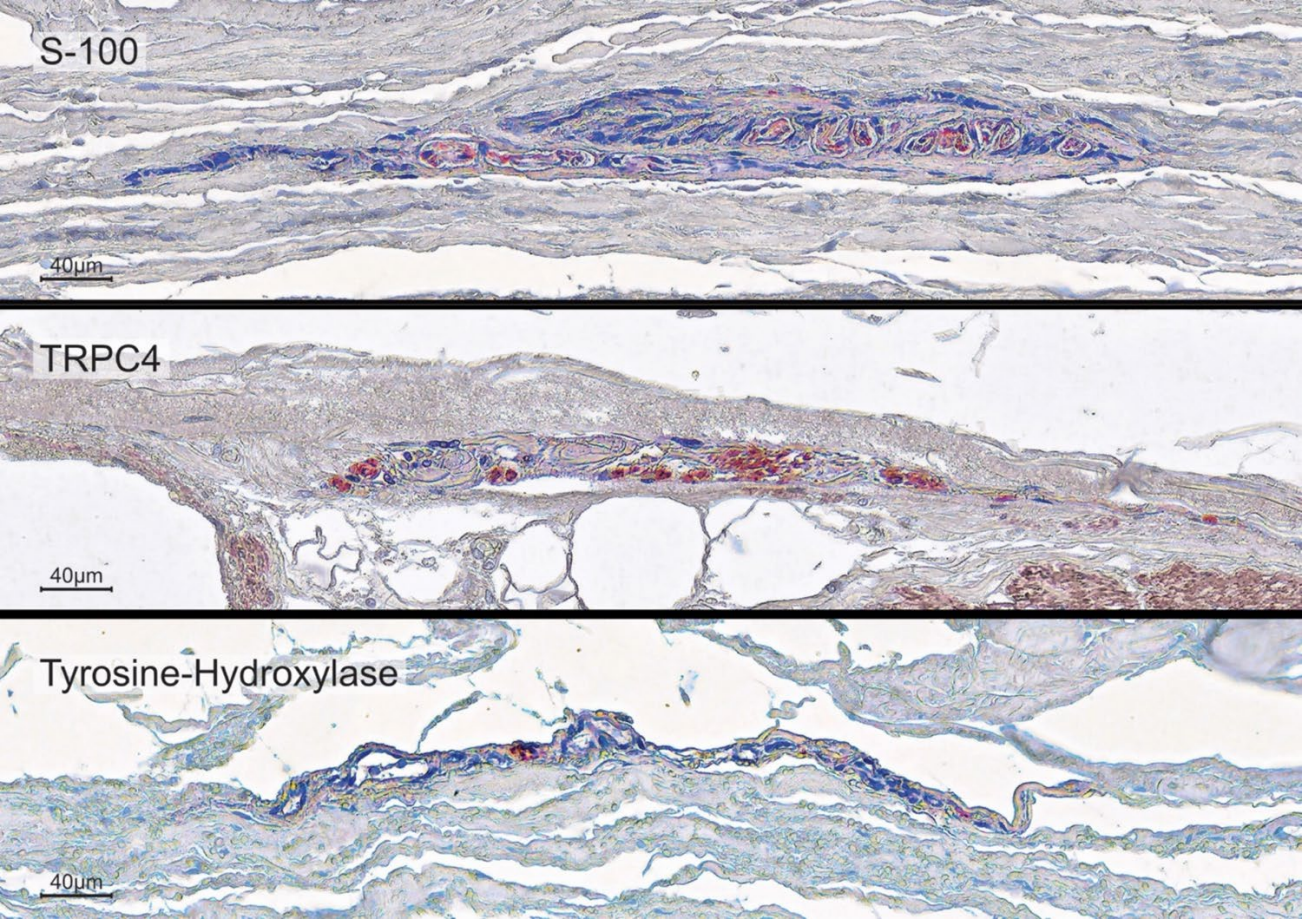
Immunohistochemical characterisation of the pericardial Ruffini-like corpuscles (RLCs). To further confirm the diagnosis of RLCs and to characterise specific neuronal components within the receptors, immunohistochemical stainings were performed using antibodies against S-100 (a specific marker for sensory fibers), Calcitonine Gene-Related Peptide (CGRP, a peptide released by sensory fibers), Transient Receptor Potential Channel 4 (TRPC4, a non-selective calcium channel of sensory fibers), and tyrosine hydroxylase (a specific marker for efferent sympathetic fibers). A positive result is indicated by the red dye, labeled on the secondary antibody. The immunohistochemistries against S-100 (top), TRPC4 (middle), and tyrosine hydroxylase (bottom) yielded positive results. Staining against TRPC4 (middle) also marked the calcium channels of the smooth muscle of all vessels. The intensity of the surrounding environment was reduced to highlight the shape and extent of the RLCs

The histochemical and immunohistochemical identification of RLCs was verified using four additional different markers that usually occur in Ruffini corpuscles, repeated ten times, following typical protocols for Ruffini corpuscles (Fig. 2). Sensory nerve fibers (S100) and nerve fibers equipped with calcium channels (TRP, specifically TRPC4) were consistently observed. An efferent sympathetic innervation (tyrosine hydroxylase) was reliably detected. Stainings for CGRP (a peptide secreted by peripheral sensory fibers) were negative.

The histological organization of the fibrous pericardium reveals three sublayers. As there is no established nomenclature for these sublayers, we propose the following labels: the outermost layer facing the thoracic cavity will be referred to as the lamina externa, the inner layer adjacent to the pericardial cavity as the lamina interna, and the layer between the two as the lamina intermedia.

**Fig. 3:**
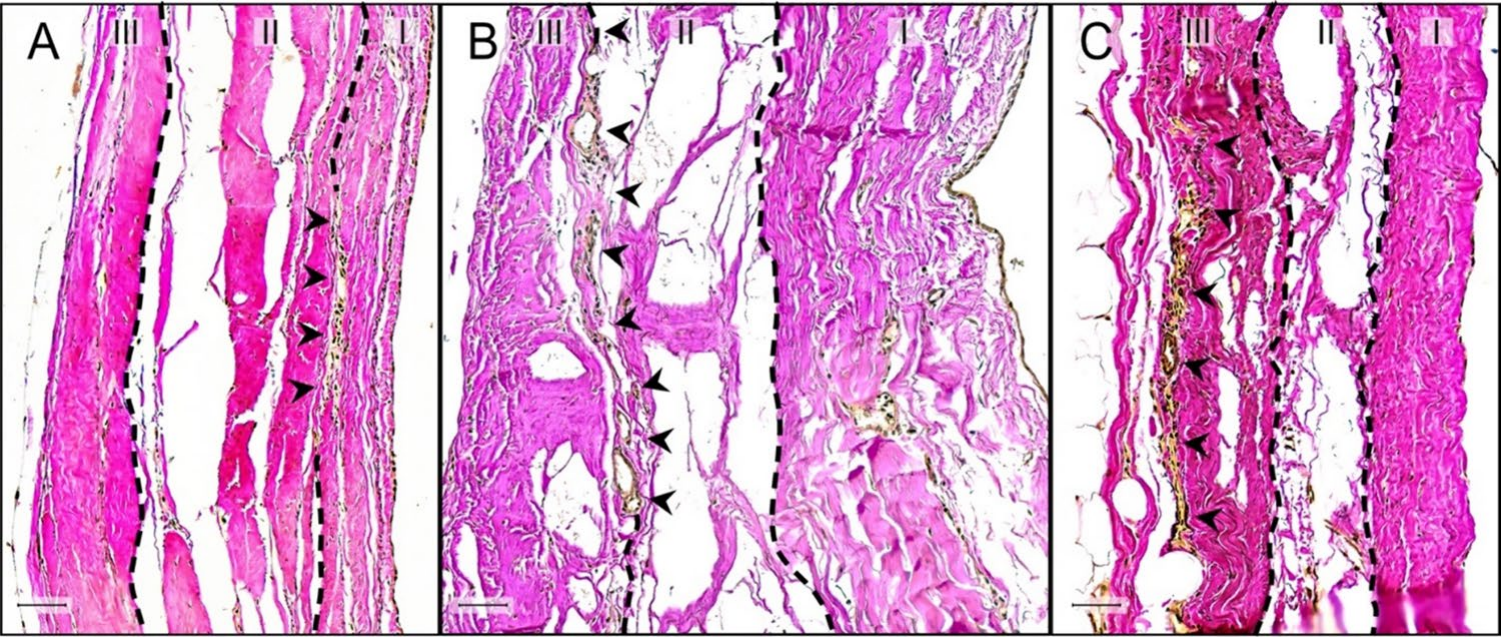
Localization of Ruffini-like corpuscles (RLCs) within the fibrous pericardium. Classification of the localization of RLCs is based on the histological organization of the human fibrous pericardium, which can be divided into three sublayers of collagenous fibers interwoven with elastic fibers. By our definition, the pericardial sublayer facing the thoracic cavity is the lamina externa (III), the middle sublayer is the lamina intermedia (II), and the sublayer touching the pericardial cavity is the lamina interna (I). The histological structure of RLCs in all localizations was identical. Receptors outside the fibrous portion of the pericardium were not considered. **A** Histological image (section thickness: 7 μm; stain: Elastica van Gieson; scale bar: 50 μm) of the pericardium of an 87-year-old female body donor. The 1st receptor localization is between the lamina interna (I) and the lamina intermedia (II) of the pericardium. Arrowheads point to the RLC positioned between these layers. Receptors of this localization are rare (12.5%). **B** Histological image (section thickness: 7 μm; stain: Elastica van Gieson; scale bar: 50 μm) of the pericardium of an 80-year-old male body donor. The 2nd receptor localization is between the lamina intermedia (II) and the lamina externa (III) of the pericardium. Arrowheads direct to the RLC at this location. This receptor localization is most abundant (45.7%). **C** Histological image (section thickness: 7 μm; stain: Elastica van Gieson; scale bar: 50 μm) of the pericardium of a 70-year-old male body donor. The 3rd localization is summarized by RLCs mainly as part of the outer border of the lamina externa (III), touching the thoracic cavity. Arrowheads indicate the RLC inside the outermost pericardial sublayer. RLCs at this spot were detected second most (41.8%)

We categorized three localization classes of RLCs according to the tripartite histological organization of the fibrous pericardium (Fig. 3). The 1st receptor localization is between the lamina interna and the lamina intermedia, while the 2nd one is positioned between the lamina intermedia and the lamina externa. The 3rd localization is summarized by RLCs mainly as part of the outer border of the lamina externa, touching the thoracic cavity. The histological structure of RLCs was uniform through all localizations (Fig. 3). Receptors between the lamina intermedia and lamina externa were most abundant (total: 674 (45.7%)), closely followed by those as part of the lamina externa (total: 617 (41.8%)). However, RLCs between the lamina interna and lamina intermedia were definitely rare (total: 185 (12.5%)).

RLCs were found in comparable proportions in both sexes (female: 703/1476 (47.6%), male: 773/1476 (52.4%)). Using scaled photographs and calculations, we approximated the mean pericardial surface of our body donations to be about 186 cm^2^ in females and 184 cm^2^ in males. This estimation aligns with the results measured by Hort [31]. There is no significant difference between the sexes regarding the dependency of corpuscles on the total pericardial surface.

**Fig. 4:**
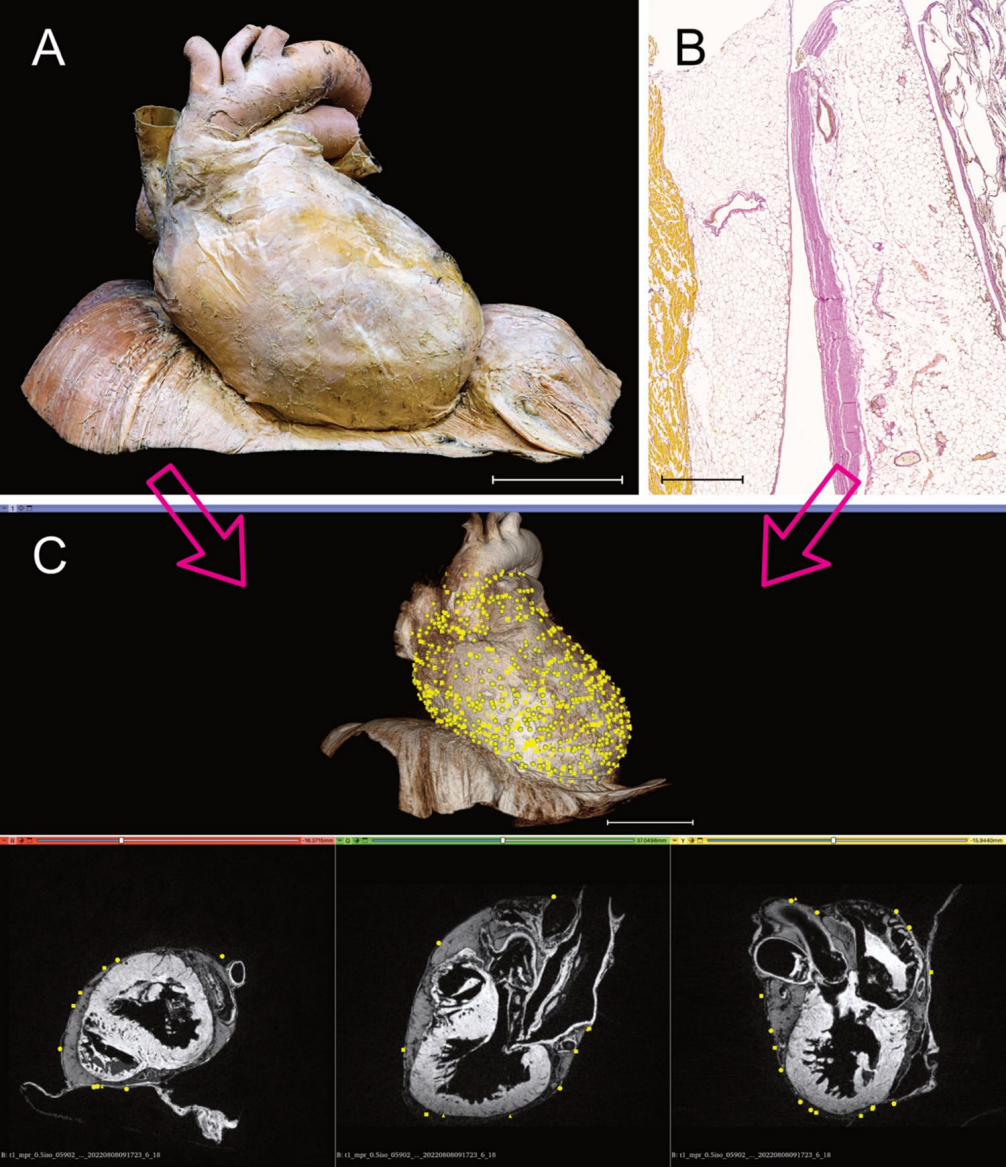
Visualization of Ruffini-like corpuscles (RLCs) in the human pericardium. To visualize the distribution of RLCs, all receptors of the eight pericardia examined were matched to a “standard-pericardium”. Using the histologically detected corpuscles with the adjacent environment and magnetic resonance imaging of the “standard-pericardium” in all three spatial planes, the receptors were transferred to the “standard-pericardium” by matching prominent anatomical landmarks. **A** Anatomical preparation of the heart within pericardium dissected from a 91-year-old formalin-fixed female body donor, which was utilized as the “standard-pericardium”. All tissue around the pericardium was carefully removed to maintain the heart’s natural in situ position on top of the diaphragm. An epoxy resin frame ensured the natural form of the diaphragm, whereas hard fixation and filling stabilized the heart within its pericardium to allow radiological 3D reconstruction by MRI and photogrammetry. Scale bar: 5 cm. **B** Exemplary histological image (section thickness: 7 μm; stain: Elastica van Gieson; scale bar: 800 μm) of the pericardium of an 80-year-old male body donor. All discovered RLCs within the pericardium (center) were matched to the corresponding position on the “standard-pericardium,” considering the myocardial topography (left) and the surrounding tissue (right). **C** Radiological volume rendering model of the “standard-pericardium” (top), to which all RLCs were assigned using the 3D Slicer program. The three forms of the yellow dots represent the different localizations of the RLCs within the fibrous pericardium (triangle = 1st localization, circle = 2nd localization, quadrate = 3rd localization). The three spatial planes included in the radiological sequence (bottom) were used for orientation in the “standard-pericardium” to achieve the most anatomically correct transfer of the receptors. Scale bar: 5 cm

**Fig. 5:**
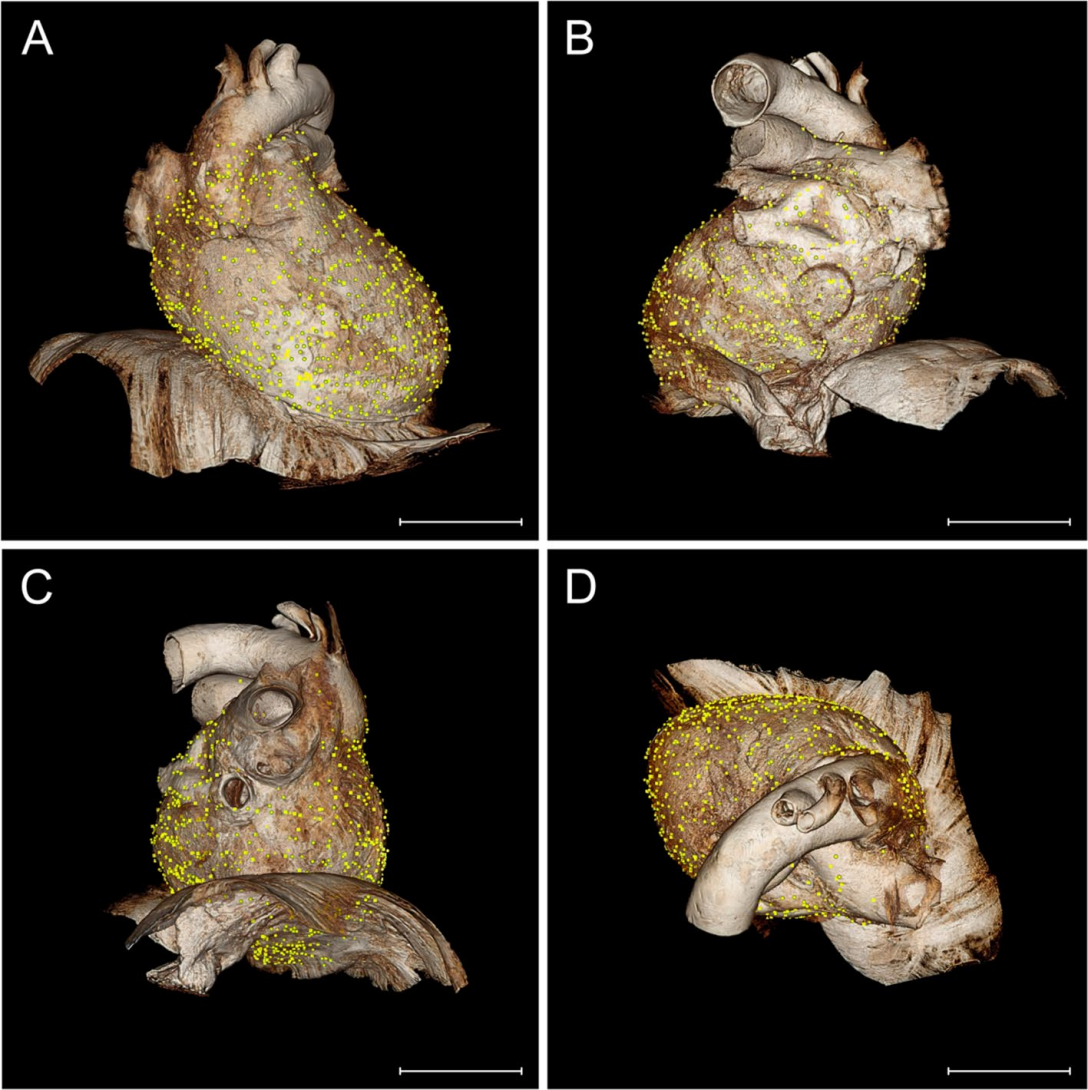
Distribution of Ruffini-like corpuscles (RLCs) in the human pericardium. Mapping and visualization of the proprioceptors were performed by digitally transferring all RLCs of the eight pericardia studied to a three-dimensional volume-rendering model used as a "standard- pericardium”. This three-dimensional model was acquired by magnetic resonance imaging from a formalin-fixated heart, including the pericardium and diaphragm of a 91-year-old female body donor. Transfer of all receptors was implemented using 3D-Slicer software. Each receptor location was mapped according to anatomical landmarks and relative to size, considering the adjacent environment and cardiac topography. Symbols represent the three pericardial localizations of corpuscles (triangle = 1st localization, circle = 2nd localization, quadrate = 3rd localization). A total number of 1476 RLCs was detected. Scale bars: 5 cm. **A** Distribution of RLCs as seen from the front of the pericardium. **B** Distribution of RLCs as seen from the posterior side of the pericardium. **C** Distribution of RLCs as seen from the left and lower sides of the pericardium. **D** Distribution of RLCs seen from above the pericardium

Each receptor position was accurately transferred to the "standard pericardium" (Fig. 4). Topographical differences with potential hotspots were already evident when all pericardia were matched to the volume rendering model (Fig. 5). Cluster analysis identified possible partitions and higher density areas within the pericardial proprioceptors.

**Fig. 6:**
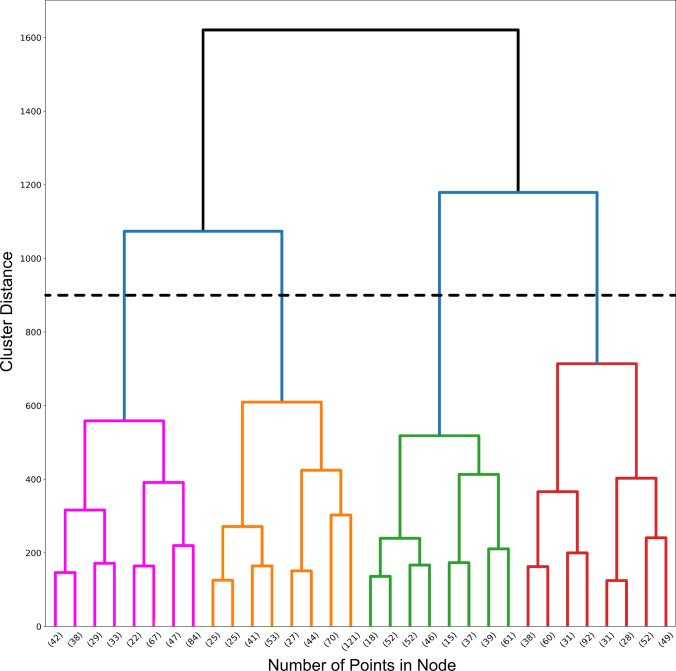
Dendrogram of hierarchical agglomerative clustering. A dendrogram partitioning the data in a logical distribution of Ruffini-like corpuscles using hierarchical agglomerative clustering with WARD linkage. We chose a cluster number of four based on the dendrogram, as indicated by the black dashed line. The x-axis displays the number of points in a given node, while the y-axis shows the cluster distance

**Fig. 7:**
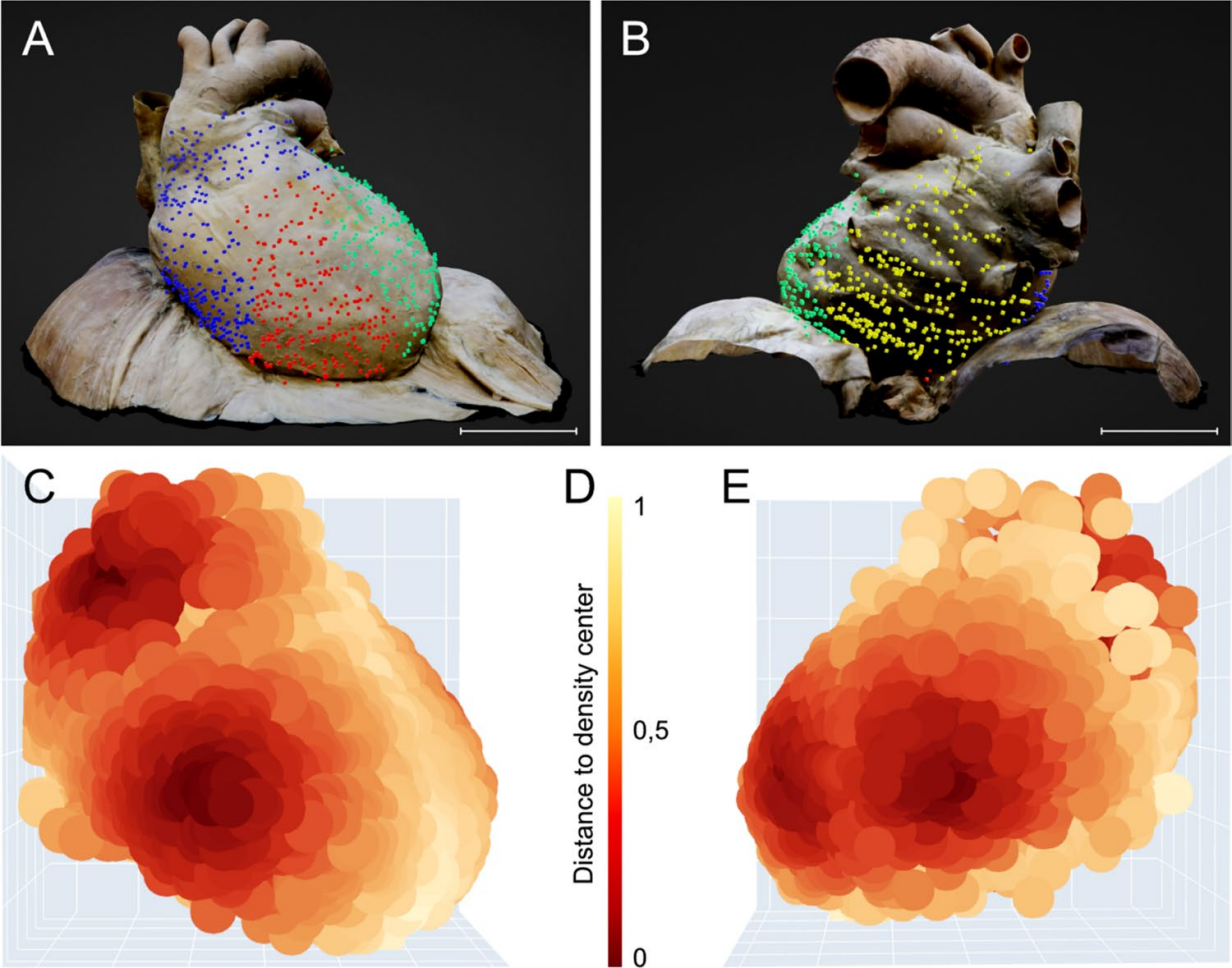
Cluster analysis of possible partitions and hotspots within the pericardial proprioceptors. Based on their relative coordinates, proprioceptors were divided into prospective partitions and areas of higher density using cluster analysis. The Python Scikit-learn package was utilized for this analysis, while the Plotly module was used for the 3D scatter plots. We used mean shift clustering in addition to the hierarchical method to find the local maxima of neighborhood density. Cluster analysis found four potential partitions and regions of increased density in the pericardial proprioceptors. The arrangement of pericardial receptor partitions correlates to the compartmentalization of the heart into four different chambers (blue: right atrium, red: right ventricle, green: left ventricle, yellow: left atrium). Hotspots of high and low receptor density within the pericardium were discovered by Mean shift function analysis of the created heatmap. Four hotspots of pericardial proprioceptors are exposed according to the four selected clusters. **A** “Standard pericardium” shown from a ventral perspective, displaying four partitions of pericardial proprioceptors determined through cluster analysis (blue = right atrium, red = right ventricle, green = left ventricle). The interventricular septum separates the two ventricles (red and green clusters), while the valvular plane separates the atria from the corresponding ventricles (blue and red clusters and green and yellow clusters (not visible from this side). Scale bar: 5 cm. **B** “Standard pericardium” viewed from a dorsal perspective with four partitions of pericardial proprioceptors identified by cluster analysis ((blue = right atrium, green = left ventricle, yellow = left atrium). The valvular plane separates the left atria (yellow) from the corresponding left ventricles (green). Scale bar: 5 cm. **C)** Heat map of the distribution of pericardial proprioceptors from a ventral perspective. Two hotspots of high receptor density are evident: at the exit of the aorta and pulmonary trunk and the right ventricular bulge, where the cardiac radius is widest. A low receptor density is found along the interventricular septum and at the cardiac apex. **D** Legend of the heat map shown in **C** and **E**. The legend displays the distance to the density center. **E** Heat map of the distribution of pericardial proprioceptors from a dorsal perspective. Two more hotspots of high receptor density are distinct: at the left ventricular bulge, where the cardiac radius is widest, and in the area of the left atrium touching the esophagus

We identified a cluster count of four and illustrated the distinct clusters as points of divergent color in a 3D scatterplot (Figs. 6 and 7A, B). One cluster (blue) corresponds to the outflow of the aorta and pulmonary trunk, as well as the right atrium. A second cluster (red) is located in the region of the right ventricle. A third cluster (green) comprises the left ventricle area and a small section of the left atrium. The remaining cluster (yellow) covers most of the left atrium, including the pulmonary veins. The interventricular septum roughly delineates the boundary between the right ventricle (red cluster) and left ventricle (green cluster). The valvular plane approximately corresponds to the border between the right atrium (blue cluster) and right ventricle (red cluster), as well as between the left ventricle (green cluster) and left atrium (yellow cluster).

Analysis of the generated heatmap using the Mean shift function reveals hotspots of both high and low receptor density within the pericardium (Fig. 7C, E). A quantile of 0.1 resulted in four clusters with a distribution similar to the hierarchical approach: First, at the exit of the great vessels (mainly the aorta and partly the pulmonary trunk). Second, at the bulge of the right ventricle (at the largest circumference of the heart). Third, at the bulge of the left ventricle (at the largest circumference of the heart). Fourth, in the region of the left atrium close to the diaphragm where the esophagus touches the pericardium. In contrast, regions of very low pericardial receptor density are located along the interventricular septum and the apex of the heart. The portions of the pericardium covering the remaining heart regions exhibit moderate receptor density.

Analysis of the generated heatmap using the Mean shift function reveals hotspots of both high and low receptor density within the pericardium (Fig. 7C, E). A quantile of 0.1 resulted in four clusters with a distribution similar to the hierarchical approach: First, at the exit of the great vessels (mainly the aorta and partly the pulmonary trunk). Second, at the bulge of the right ventricle (at the largest circumference of the heart). Third, at the bulge of the left ventricle (at the largest circumference of the heart). Fourth, in the region of the left atrium close to the diaphragm where the esophagus touches the pericardium. In contrast, regions of very low pericardial receptor density are located along the interventricular septum and the apex of the heart. The portions of the pericardium covering the remaining heart regions exhibit moderate receptor density.

## Discussion

The objective of this study was to monitor proprioceptors in the human pericardium. RLCs were the only corpuscles detected in substantial quantities. According to their position within the three pericardial sublayers, the RLCs were assigned to three localization classes (Fig. 3). We were able to verify the tripartite histological organization of the fibrous pericardium in all specimens studied in accordance with the literature [29, 30, 73]. Most RLCs were located between the lamina intermedia and lamina externa (class 2), and second most at the outer border of the pericardium within the lamina externa (class 3). We transferred the proprioceptors detected in the fibrous pericardial portions of all eight pericardia to macroscopic models (Fig. 5). Cluster analysis revealed the highest receptor density in the region of the ventricular bulges and at the outlet of the great vessels (Fig. 7). It can be inferred from these findings on pericardial propriosensing that displacements within the fibrous pericardial sublayers (class 1 and 2) and expansions at the outer pericardial border to the environment (class 3) are registered. These relative movements are affected by cardiac parameters, such as heart rate, contraction/relaxation, volume shifts (end-systolic volume, end-diastolic volume), and ejection fraction. Besides, even higher level perceptions such as time awareness [3] could be deduced.

In the musculoskeletal system, Ruffini corpuscles primarily register stretching, i.e., displacements or movements of tissue layers relative to each other [9, 27]. However, nothing can be found on their function in the viscera. Because they have yet not been studied electrophysiologically in internal organs, their relevance remains unclear and can only be assumed by analogies [75]. To date, no mechanoreceptors have been studied directly and precisely in either animal or human pericardium. We detected most receptors between the lamina intermedia and lamina externa and within the lamina externa bordering the thoracic cavity. Consequently, local gliding is likely to be particularly important at these sites, whereas the small number of receptors found between the lamina interna and lamina intermedia probably reflects a subordinate role in relative movements of the pericardial sublayers. Furthermore, this arrangement allows distinguishing displacements between the outer fibrous pericardial sublayers, i.e., of the pericardium itself and displacements of the pericardium against the adjacent tissues. We suggest that Ruffini corpuscles primarily register displacements between pericardial sublayers and between the pericardium and its adjacent structures. Yet, interpretation of the importance of the localization classes is limited as detailed biomechanical studies on the significance of the three pericardial sublayers are lacking.

As documented in the literature [58], we confirmed the sympathetic efferent innervation (tyrosine hydroxylase) of the RLCs, presumably allowing for regulatory control of the response thresholds to adrenergic stimuli. The significance of autonomic innervation of RLCs in the musculoskeletal system is poorly understood. Therefore, no direct analogies can be drawn regarding lowering or raising thresholds under increased sympathetic tone. Nevertheless, threshold rise has been documented for sympathetic activation, lowering pain perception [14]. Overall, it is believed that RLCs are at least involved in pain perception [64]. This is confirmed by the presence of Ruffini corpuscles alongside nociceptive fibers [34, 45], suggesting a potential indirect role in modulating pain through proprioceptive feedback. However, in accordance with others, we could not demonstrate CGRP-positive fibers in RLCs [65], which are typically involved in pain perception [32].

Kostreva and Pontus [37] also reported mechanoreceptor conduction in the canine pericardium. However, unlike our findings in the human pericardium, these receptors are concentrated along the atria and atrioventricular grooves. We observed that receptors were concentrated in the area of the ventricular bulges and at the outlet of the great vessels. Evolutionarily, this discrepancy may be attributed to the bipedal walk. Human’s erect posture causes a significant amount of blood volume to enter the heart following the force of gravity and be ejected upward against the force of gravity. This results in different volume dynamics compared to pumping blood perpendicular to gravity. Furthermore, the human heart is more suspended on its great vessels due to upright gait, while in quadrupeds, it resides primarily on its superior or lateral ventricular walls. Besides, Lee and Boughner [41] demonstrated that there are discrepancies in the thickness and viscoelastic properties of the human and canine pericardium. Consequently, the animal model's validity for humans is restricted.

The knowledge regarding the mechanoreceptors in the pericardium of animals is notably limited. Morphological and electrophysiological studies have identified nerve fibers or plexuses in a few animals, which suggests a mechanoreceptive function of the pericardium [18, 37, 61]. Some studies have investigated the potential role of mechanosensors in the pericardium and the activities of proprioceptive neurons in the spinal cord and cortical regions in processing mechanoreceptive information [43]. However, as we only identified studies that were likely derived from RLC-equipped pericardia of dogs or guinea pigs, it has to be stated that there are not sufficient morphological studies to improve functional understanding by comparative anatomy or evolutionary analyses. Nevertheless, the impact of these control circuits on the afferent side on heart dysfunctions offers interesting possibilities for therapy.

Our cluster analysis revealed four potential partitions and four hotspots of high density among the pericardial proprioceptors (Fig. 7). The clustering of pericardial receptor partitions corresponds to the heart's compartmentalization into four distinct chambers (in Fig. 7A, B, blue = right atrium, red = right ventricle, green = left ventricle, yellow = left atrium). Cluster boundaries are located at the anatomical borders of the heart between the four chambers. The interventricular septum separates the two ventricles (red and green cluster), while the valvular plane isolates the atria from the corresponding ventricles (blue and red cluster and green and yellow cluster). Although we see four separate measurement zones, it can be assumed that shifts in the pericardium at one location lead to signaling at the others.

Four pericardial proprioceptor hotspots (Fig. 7C, E) are exposed using the ward function. A prominent cluster of RLCs is located at both ventricular bulges, where the radius perpendicular to the longitudinal axis of the heart is the widest, indicating the outermost extension of the ventricles. Additionally, receptors accumulate at the outlet of the great vessels, particularly around the ascending aorta, where the visceral sheet of the serous pericardium transitions to the parietal sheet. These regions undergo significant volume changes during the cardiac cycle. Pronounced volume-related stretching of the pericardial layers is, therefore, sensed by a particularly large number of RLCs, emphasizing the most robust signals. For the hotspot surrounding the outlet of the great vessels, the fluctuation in diameter of the aorta and the pulmonary trunk (i.e., Windkessel function) must be considered. Volume fluctuations during swallowing may explain the additional receptor hotspot in the region of the left atrium bordering the esophagus. The low receptor density in the region of the interventricular septum may be attributed to its extraordinary role in the biomechanics of the cardiac cycle [68], whereas the missing receptors in the apex may be due to its limited exposure to volumetric changes, as it remains more stationary during the cardiac actions of the ventricles [28]. Accordingly, monitoring cardiac volume changes through pericardial proprioceptors does not appear relevant at these sites.

Sherrington [60] described proprioceptive “reflex arcs”, in which a motor response was observed as a feedback mechanism to a proprioceptive stimulus. Tuthill and Azim [69] reviewed the existence of supraspinal ascending proprioceptive pathways, in addition to the basic spinal reflex pathways that directly signal from proprioceptors to interneurons and motoneurons in the spinal cord. Ruffini corpuscles might serve as an external control mechanism capable of perceiving the stretching of the heart during cardiac cycle. However, proprioceptors in the human pericardium cannot provide the same level of control arc as extrinsic reflexes of skeletal muscles, as the heart lacks direct neuronal motor excitation. Nonetheless, proprioceptors likely modulate various cardiac parameters. It is generally accepted that the autonomic nervous system has an influence on cardiac output [11, 54]. RLCs detect shifts between pericardial sublayers and could be relevant for cardiac feedback by modulating the sympathetic or parasympathetic nervous system. Proprioceptive monitoring may, therefore, lead to changes in dromotropy, lusitropy, chronotropy, inotropy, and bathmotropy, either individually or in any combination. However, relative to each other, shifts of the pericardial sublayers depend on volume changes in time, location, and rate. As a result, Ruffini corpuscles would primarily provide information on heart rate (chronotropy) and stroke volume (inotropy and lusitropy).

Potentially disturbed pericardial feedback could play a critical role in the pathogenesis of several diseases. Changes in movement and distension of pericardial regions may cause significant alterations in sensory signaling. This could, for example, be attributed to an increase in the amount of pericardial fluid, such as in cases of pericardial serous effusion or hemorrhage. In such instances, the rapidly developing hemorrhage leads to greater receptor excitement resulting from the fast displacement of pericardial sublayers than it would occur during a slower increase in serous fluid production. On the other hand, an existing effusion, by utilizing the stretching reserve combined with a smaller extension of the heart, leads to fewer displacements of the pericardial layers. This will likely result in less contrasted feedback, negatively impacting cardiac regulation. The subsequent inadequate proprioceptive feedback may contribute to palpitations.

Fibrotic remodeling of the pericardium following pericarditis or intervention-induced scar formation reduces elasticity, causing the pericardial sublayers to adhere to one another. This adhesion limits or entirely prevents the expansion and relative sliding of the pericardial sublayers. Consequently, the afferent limb of the pericardial loop becomes inadequate.

Disrupting the pericardium’s integrity, such as in pericardial opening during surgical procedures or pericardiectomy, may lead to decreased sensitivity. Particularly pericardiectomy, implemented as a treatment for pericarditis, is associated with high perioperative and long-term mortality rates [4, 20, 46]. This may indicate that the pericardium performs mechanical functions and is also involved in feedback to monitor cardiac function. A resection of the pericardium or cases where the pericardium is left open could lead to a partial or complete loss of the afferent proprioceptive pericardial limb. This might be associated with life-threatening symptoms.

It is relevant to note that in the context of pericardial diseases in the scientific literature, the pathophysiology is mainly reduced to mechanical consequences, and others, e.g. sensory functions, are not taken into account. This may be due to the lack of information about sensory feedback from the pericardium. However, it is crucial to consider impaired regulation as another relevant factor in the pathophysiology of constrictive pericarditis, cardiac tamponade, and pericardial integrity disturbances. This perspective also suggests potential avenues for therapeutic interventions [2].

The use of pericardial tissue exclusively from body donors, with an average age of 82 years, led to limitations of this study. Potentially, the number and distribution of RLCs may differ in younger age cohorts and change with aging. It has been observed in both human and animal musculoskeletal systems that the number of proprioceptors tends to decline with age [5, 42, 44]. However, Ruffini corpuscles have a unique capacity for regeneration [77]. Therefore, objective evaluations of the receptor number in younger populations and their possible dynamics cannot be conducted. Furthermore, only RLCs can be accurately identified when their fusiform shape is cut longitudinally parallel to the orientation of the collagen. It is plausible that there may exist corpuscles running vertically between the pericardial sublayers, but their identification remains uncertain. Since the extent of this component is unclear, we have refrained from extrapolating the total amount of RLCs to the entire pericardial surface. Despite the limited number of pericardia, we observed clear foci in the receptor distribution that were consistent throughout all specimens. Because of the limited comparable literature, there is no further support for explaining this striking spatial distribution. These observations thus serve as a foundation for following functional studies and provide a suitable basis for digital experiments.

Our study demonstrated the existence of RLCs within the fibrous pericardium. Confirmation with other methods, such as electrophysiological studies or evidence of feedback mechanisms, would be required for further evidence. However, the high number of receptors suggests significant implications. Therefore, we suggest that pericardium serves an undiscovered function as a sensor with the RLCs as its anatomical correlate.

### Essentials

(1) RLCs are present in large numbers in the human pericardium, (2) Based on the tripartite histological organization of the human pericardium, they can be classified into three localization groups, (3) The receptor distribution follows a distinct pattern with highest density in the region of the largest ventricular diameter and at the transition to the large vessels, and (4) No sex-specific differences in the number or distribution of receptors can be detected.

Inferential, the human pericardium is subject to neural proprioceptive control.

## Data Availability

We can provide datasets for 3D data (3D-Slicer), statistics (SPSS), and visualization of cluster analysis (HTML).
